# Supplementary material for: Causal Relationship between Aging and Anorexia Nervosa: A White-Matter-Microstructure-Mediated Mendelian Randomization Analysis
Source: Biomedicines. 2024 Aug 16;12(8):1874. doi: 10.3390/biomedicines12081874 (PMC11351342; doi:10.3390/biomedicines12081874)
Supplement: Supplementary file 1 [file biomedicines-12-01874-s001.zip › Supplementary Figure/Figure S9/Leaveoneout plot of MR analysis for ubm-b-1843 to AN.pdf]

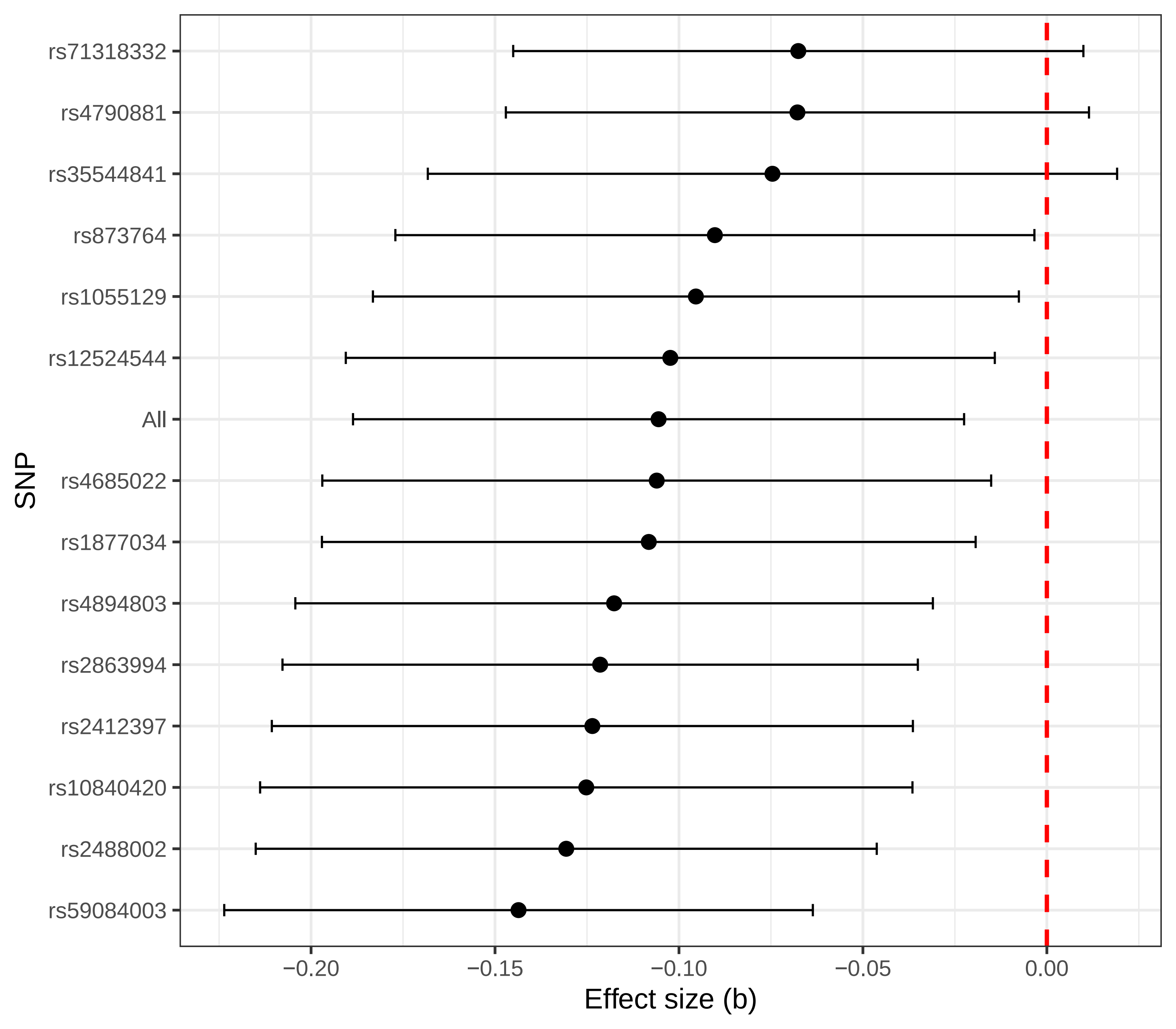
rs71318332
rs4790881
rs35544841
rs873764
rs1055129
rs12524544
All
rs4685022
rs1877034
rs4894803
rs2863994
rs2412397
rs10840420
rs2488002
rs59084003
SNP
−0.20
−0.15
−0.10
−0.05
0.00
Effect size (b)
